# Embracing novel thinking to safeguard against airborne pathogens in indoor spaces

**DOI:** 10.5588/ijtldopen.24.0198

**Published:** 2024-06-01

**Authors:** H. van der Westhuizen, J.-A. Nice, C. Tudor, Y. Liu, S. Ahmedov, A.R. Kansal, P.A. Jensen, R.L. Vincent, G. Mustapha, V. Vauhkonen, I.M. Ochoa Delgado, M. van der Walt, G. Volchenkov

**Affiliations:** ^1^ End TB Transmission Initiative – Powering Airborne IPC, Working Group of the Stop TB Partnership, Geneva, Switzerland;; ^2^ Nuffield Department of Primary Care Health Sciences, Oxford University, UK;; ^3^ TB Proof, Cape Town, South Africa;; ^4^ Independent TB IPC Built Environment Consultant, Tshwane, South Africa;; ^5^ Department of Architecture & Industrial Design, Tshwane University of Technology, Tshwane, South Africa;; ^6^ Johns Hopkins University School of Nursing, Baltimore, MD, USA;; ^7^ Clinical Center on Tuberculosis, Beijing Chest Hospital, Beijing, China;; ^8^ Bureau for Global Health, TB Division, United States Agency for International Development, Washington DC, USA;; ^9^ National Institute of TB and Respiratory Diseases, New Delhi, India;; ^10^ Final Approach Inc, Port Orange, United States of America;; ^11^ Icahn School of Medicine at Mount Sinai, New York City, USA;; ^12^ KNCV Tuberculosis Foundation, The Hague, The Netherlands;; ^13^ Independent Heating, Ventilation and Air Conditioning Consultant, Helsinki, Finland;; ^14^ Hospital Nacional Docente Madre Niño San Bartolome, Lima, Peru;; ^15^ Tuberculosis Platform, South African Medical Research Council, Pretoria, South Africa;; ^16^ Independent TB and Airborne IPC Consultant, Vladimir, Russia

**Keywords:** infection prevention and control, tuberculosis, COVID-19, airborne disease

The COVID-19 pandemic focussed global attention and resources on airborne infection prevention and control (IPC). This has stimulated advances in the field that have universal application to other airborne pathogens such as TB, influenza, and measles. We argue that it is important to sustain the momentum in these advances, not only to keep indoor spaces safe from known airborne pathogens but to also ‘pandemic proof’ shared spaces, including health care settings. This should be combined with the systematic approach recommended by the WHO for TB IPC, which is also relevant to airborne disease in general. Here, we review airborne IPC research that occurred during the COVID-19 pandemic and highlight four areas: political prioritisation; ventilation and novel technologies; shared lessons between COVID-19 and the TB response; and advances in personal protective equipment (PPE).

## Political prioritisation for clean indoor air and best practice guidelines for buildings

WHO guidelines released in 2021 aimed at improving indoor air ventilation to extract and dilute SARS-CoV-2, with guidance for people isolating at home, health care settings and other congregated indoor settings.^1^ In the United States, a Clean Air in Buildings Challenge led to federal funds being used to improve indoor air quality, including $350 billion allocated for state and local governments, as well as $122 billion for schools.^2,3^ In South Africa, the minimum ventilation standard in national building regulations was updated, shifting from a sole focus on the number of air changes per hour, to one that also considers the size and occupancy of the space, which was also reflected in 2021 COVID-19 guidance for health facilities.^4^ In India, the Right to Information Act was used to evaluate the safety of indoor air in key public buildings, also highlighting the importance of making this information visible to the public.^5^ These examples form part of a global shift towards recognising the broad value offered by investments in transmission prevention and improved air quality, which should be further encouraged.^6^

## Indoor ventilation and novel technologies

A long-standing challenge with airborne IPC has been making an invisible risk, measurable and actionable. The COVID-19 pandemic triggered widespread use of carbon dioxide (CO_2_) devices that display the fraction of rebreathed air in a closed setting, which can be used as a proxy for airborne pathogen transmission risk. This has also been of interest in TB research, but it is not widely utilised in clinical care.^7^ The use of CO_2_ devices during the COVID-19 pandemic included citizen advocacy (see the online group ‘CO_2_ guerillas’) and monitoring the safety of air in schools.^8^ This approach provides advantages when compared to the episodic, paper-based checklist tools for airborne IPC assessment – primarily that it can drive improvements in real time and capture longitudinal data. It can also identify trends, such as typical times during the day (or days of the month) when buildings have elevated CO_2_ levels (e.g., due to overcrowding), and aid real-time interventions through digital dashboards. However, there are limitations to using CO_2_ as an indicator of airborne pathogen transmission risk. For example, air cleaning reduces the viable pathogen concentration, whereas CO_2_ levels remain unchanged. Outdoor CO_2_ levels also affect indoor measurements. In some cases, if a person with an infectious disease comes into close contact with others, it is still possible for an airborne disease to be transmitted despite being in a well-ventilated space with low levels of CO_2_. The principle of ‘near field’ and ‘far field’ infection response is a critical aspect in logarithmic risk calculation. As with all airborne IPC measures, CO_2_ monitors should be used as part of a suite of prevention solutions rather than as a standalone tool. However, we are positive about the additional value that such a visualisation of risk demonstrates and view it as a powerful resource for advocacy and action.

## Shared learning between the TB and COVID-19 IPC communities

Standalone room air cleaners with high-efficiency particulate air (HEPA) or other high-efficiency filters were excluded from the recommendations of the 2019 WHO TB IPC guidelines, but have been included in the 2021 package developed by WHO for COVID-19.^1,9^ Similarly, germicidal ultraviolet (GUV) is recommended in WHO guidelines for TB, but not COVID-19.^1,9^ Both of these systems could fill a gap that health workers in high-TB burden countries have long identified: infrastructure changes to improve building design take time, are not always practical, and involve multiple budgetary processes. During the COVID-19 pandemic the ‘do-it-yourself’ Corsi-Rosenthal box was used as a low-cost project to simultaneously improve ventilation and air-cleaning in classrooms and engaged a broader group of people in improving indoor air quality.^10^ Groups like Clean Air Stars developed practical guidance for choosing room air cleaners, accounting for acceptable noise levels and room occupancy.^11^ GUV systems have a strong evidence base for TB and offer significant promise as universal airborne IPC measures.^12^ Importantly, both room air cleaners and GUV systems require careful regulation and expert guidance on placement, installation, and maintenance planning. We believe that there are opportunities for shared learning between the COVID-19 and TB IPC communities on the effective and safe use of these tools and to jointly focus on preventing the transmission of airborne pathogens.

## Advances in personal protective equipment

The COVID-19 pandemic evoked a global response to the benefit of masking and likely led to reduced stigmatisation of mask-wearing for other diseases such as TB.^13^ However, the risks faced by essential workers during the COVID-19 pandemic highlighted significant design flaws in personal respiratory protection. A systematic review found that fit test pass rates were lower among females and people of black, Asian and minority ethnic groups, which form considerable parts of the global health workforce.^14^ The significance of COVID-19 transmission in schools also meant that respiratory protection for children and adolescents was required, but appropriate smaller designs were not readily available.^15^ The limited range of available PPE has important implications for its use in TB, where an adequate seal is important and healthcare workers often wear the respirator for extended periods of time and need to communicate through it. National TB programmes should opt for respirator designs that have a higher fit test pass rate for their local population and offer various respirator designs and sizes.^16^ We anticipate that the common practice of using respirators during the COVID-19 pandemic would make the use of well-fitting PPE more acceptable in areas with a risk of airborne diseases. We also view a greater variety of respirator designs as a positive step towards offering more reliable and protective options to health workers and communities alike.

## CONCLUSION

The COVID-19 pandemic demonstrated the value of interdisciplinary airborne IPC to rapidly respond to outbreaks of airborne pathogens. It is important to sustain this and safeguard health facilities and shared indoor spaces as a component of pandemic preparedness. Traditional hospital-based IPC has typically targeted measures towards people displaying symptoms of a respiratory illness. COVID-19 has shown the importance of early intervention when a person is still asymptomatic. This also applies to TB, where asymptomatic and pre-symptomatic transmission is likely, but further research is needed.^17^ Considering airborne disease prevention broadly, we believe that this should prompt a paradigm shift in our thinking with increased investment in universal airborne infection control measures in all high-risk spaces (including health facilities and community settings). This is a core component of pandemic preparedness and is also useful for known airborne diseases, including TB and seasonable respiratory diseases. This is an exciting time to work in the field of airborne IPC. We need to harness the latest innovations to make a tangible difference to the safety of shared indoor spaces.
